# In Vivo mRNA-Lipid Nanoparticle CAR-T Cell Engineering: Advances, Challenges, and Clinical Translation

**DOI:** 10.3390/biomedicines14061276

**Published:** 2026-06-03

**Authors:** Vipin K. Yadav, Priyanka Yadav, Sreevidya Mallappa, Praveen Neeli

**Affiliations:** 1 Department of Clinical Science, Moffitt Cancer Center, Tampa, FL 33612, USA; vipin.yadav@moffitt.org; 2 Department of Gastroenterology, Institute of Medical Sciences, Banaras Hindu University, Varanasi 221005, India; priyankay.1113@gmail.com; 3 Centre for Chemical Biology, CSIR-Indian Institute of Chemical Technology, Hyderabad 500007, India; sreevidyamallappa@gmail.com; 4 Department of Molecular Oncology, Moffitt Cancer Center, Tampa, FL 33612, USA

**Keywords:** mRNA delivery, lipid nanoparticles, CAR-T cell therapy, in vivo reprogramming, adoptive immunotherapy, gene delivery

## Abstract

Chimeric antigen receptor T (CAR-T) cell therapy has transformed the treatment of hematologic malignancies, yet its broader application, particularly in solid tumors, remains constrained by high cost, labor-intensive manufacturing, limited production capacity, and variable clinical performance, as well as barriers such as poor trafficking, antigen heterogeneity, and an immunosuppressive tumor microenvironment. In vivo CAR-T cell engineering, in which CAR-T cells are generated directly within the patient, offers a paradigm shift by eliminating the need for ex vivo cell processing and complex logistical infrastructure. Among emerging approaches, messenger RNA (mRNA)-loaded lipid nanoparticles (LNPs) have emerged as a promising and clinically tractable platform for in vivo CAR-T cell generation, enabling direct reprogramming of T lymphocytes within the patient and thereby circumventing the need for leukapheresis, viral vector production, and prolonged ex vivo culture, effectively transforming the patient into their own cell therapy factory. This review synthesizes advances in mRNA-LNP-mediated in vivo CAR-T cell generation, encompassing ionizable lipid chemistry and emerging T cell-targeted delivery strategies, including surface functionalization approaches. We discuss the implications of transient CAR expression for immune activation, safety, and therapeutic durability, alongside CAR design optimization through co-stimulatory domains and safety switches. Preclinical evidence from murine tumor models and non-human primates is integrated with current regulatory considerations, and key barriers to clinical translation are highlighted. Collectively, progress in nucleic acid delivery, synthetic immunology, and precision medicine positions in vivo mRNA-CAR-T therapy as a promising modality for oncology and beyond.

## 1. Introduction

**Figure 1:**
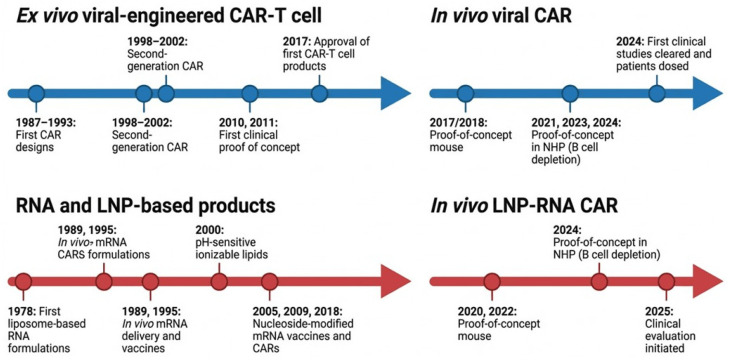
Timeline of major discoveries and milestones of CAR-T therapies leading to clinical translation of in vivo CAR technologies. The development of ex vivo-engineered chimeric antigen receptor (CAR) T cell therapies marked a breakthrough in modern medicine, but it also revealed important challenges related to accessibility and clinical performance (**top left**). To address these limitations, in vivo CAR approaches have begun to emerge, driven by advances in virology, RNA therapeutics, and nanomedicine (**bottom left**). From this convergence, two main in vivo CAR strategies have moved toward clinical translation: engineered viral vectors (lentiviral), which integrate the CAR construct into the genome for stable expression (**top right**), and lipid nanoparticle (LNP)-based RNA delivery systems, which enable transient CAR expression (**bottom right**).

Immunotherapy has undergone a major transformation over the past three decades, driven by advances in monoclonal antibodies [1,2], antibody–drug conjugates [3], cytokines [4], immune cell engagers [5], DNA- and RNA-based vaccines [6,7,8], and, more recently, engineered T cell therapies [9]. Among these modalities, autologous chimeric antigen receptor (CAR) T cell products in which a patient’s own T cells are collected, genetically modified ex vivo, and reinfused have demonstrated unprecedented potency, inducing durable remissions and apparent cures in subsets of individuals with relapsed or refractory B cell malignancies, diffuse large B cell lymphoma, and multiple myeloma [10]. Since the first approval of tisagenlecleucel (Kymriah) in 2017, CAR-T cell therapy has become a paradigm-shifting treatment for otherwise treatment-refractory hematological malignancies [11]. A historical overview of key milestones in CAR-T cell therapy development is shown in Figure 1. Despite this clinical impact, the broader use of autologous CAR-T cell therapy has been severely constrained by its manufacturing, logistical, and economic burden [12]. Current approved products require leukapheresis, cell isolation and activation, viral vector transduction, expansion, extensive quality control testing, and cryopreservation [13]. This multi-step workflow typically takes three to six weeks from leukapheresis to product release, during which patients with rapidly progressive disease may clinically deteriorate or become ineligible for treatment [14]. Per-patient treatment costs for a single course are commonly in the range of 400,000 to 500,000 USD, and manufacturing failures occur in approximately 5–10% of cases, further compounding access limitations and financial risk [15]. As a result, only a fraction of patients who might benefit from CAR-T cell therapy are ultimately treated. These constraints, together with the requirement for chemotherapy-based lymphodepletion and the risk of toxicities such as cytokine release syndrome and immune effector cell-associated neurotoxicity, have prompted intense interest in next-generation strategies that simplify or bypass ex vivo cell manufacturing [16]. One of the most promising approaches is in vivo CAR-T cell engineering using lipid nanoparticles (LNPs) loaded with mRNA to reprogram endogenous T cells directly within the patient [17]. In this paradigm, the patient’s immune system functions as a living bioreactor. LNPs deliver transient CAR-encoding mRNA to circulate T lymphocytes, which then acquire antitumor specificity without the need for cell harvesting or ex vivo manipulation [18]. This strategy builds on the same mRNA-LNP platform that enabled rapid development and global deployment of COVID-19 vaccines but repurposes it for precision immunotherapy. Recent preclinical studies have demonstrated that LNP formulations can be designed to preferentially target and transfect T cells, enabling the generation of functional CAR-T cells entirely in vivo and mediating effective antitumor responses in multiple models [19,20,21,22,23,24]. Early clinical efforts with RNA-LNP platforms and other in vivo CAR technologies suggest that efficient in situ CAR expression, rapid B cell or tumor depletion, and manageable safety profiles are achievable, establishing proof of concept for this modality [25]. In parallel, advances in mRNA chemistry, ionizable lipid design, and targeted nanomaterials are expanding the toolbox for modulating expression kinetics, tropism, and immunogenicity.

This Review focuses on in vivo mRNA-CAR-T therapy as an emerging class within the broader field of in vivo immune engineering. We first outline the molecular biology of mRNA therapeutics and principles of LNP design that are most relevant to T cell targeting. We then discuss strategies for achieving selective delivery to T lymphocytes and considerations for CAR construct optimization in the context of transient in vivo expression. Next, we summarize preclinical and nascent clinical evidence supporting in vivo mRNA-CAR-T approaches across hematological malignancies, solid tumors, and autoimmune disease. Finally, we highlight the key challenges, opportunities, and translational pathways that will determine whether in vivo mRNA-CAR-T therapy can overcome the cost, time, and failure-rate limitations of current autologous CAR-T cell products and achieve widespread clinical adoption.

## 2. mRNA Biology and LNP Delivery Platform

### 2.1. Renaissance of mRNA Therapeutics

**Figure 2:**
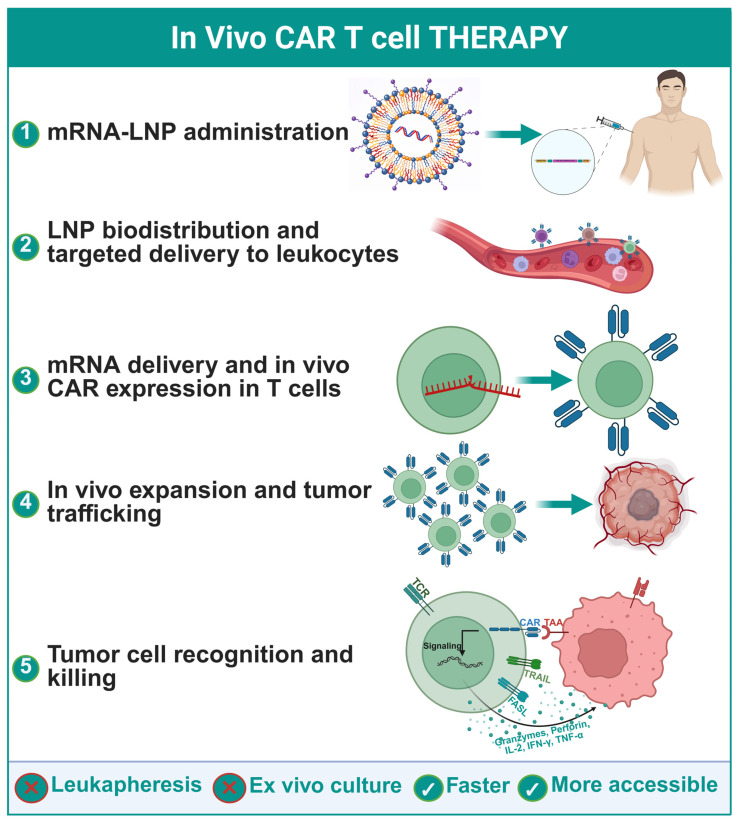
Schematic of workflow for in vivo delivery of CAR T cells using mRNA-loaded lipid nanoparticles (LNPs). Systemic delivery of LNPs enables distribution and targeting of leukocytes, mRNA delivery to T cells to express CARs, in vivo expansion and tumor trafficking, and recognition and killing of tumor cells by CAR T cells, avoiding leukapheresis and ex vivo culture for rapid and accessible treatment.

mRNA is now viewed as a versatile therapeutic modality because it can encode virtually any protein of interest while avoiding the risks associated with genomic integration [26]. However, early enthusiasm was tempered by the intrinsic instability of mRNA and its strong activation of innate immune sensors, and the challenge of delivering large, polyanionic macromolecules across cellular membranes in vivo [27]. A pivotal advance came in the mid-2000s, when Karikó, Weissman and colleagues showed that replacing uridine with modified nucleosides such as pseudouridine and, later, N1-methylpseudouridine (m1Ψ) markedly attenuates innate immune recognition while enhancing translational efficiency, thereby laying the foundation for contemporary mRNA therapeutics [28]. Modern mRNA constructs used for CAR-T cell generation incorporate several layers of sequence and structural optimization. These typically include: a synthetic 5′ cap analog that supports efficient cap-dependent translation initiation; carefully selected 5′ and 3′ untranslated regions derived from highly translated endogenous transcripts (for example, α-globin or Xenopus β-globin) to promote stability and ribosome loading; codon optimization of the open reading frame to align with human codon usage and minimize inhibitory sequences; a poly(A) tail of approximately 100–150 nucleotides to enhance stability and facilitate ribosome recycling; and systematic incorporation of modified nucleosides, particularly m1Ψ, to dampen Toll-like receptor signaling and reduce type I interferon induction [29,30]. Collectively, these engineering strategies extend the effective half-life of therapeutic mRNA from minutes to hours or days and substantially increase the amount of protein produced per molecule delivered, properties that are critical for efficient in vivo CAR expression. An overview of the in vivo CAR-T cell engineering strategy using mRNA-LNP delivery is illustrated in Figure 2. 

### 2.2. Lipid Nanoparticle Architecture

**Figure 3:**
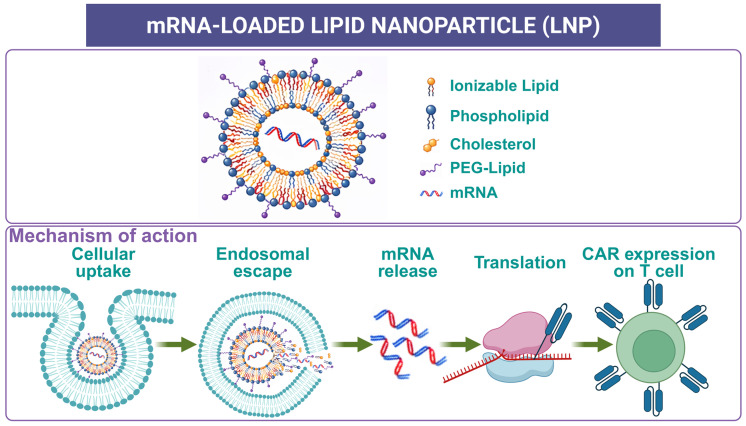
Structure and mechanism of mRNA-loaded lipid nanoparticles (LNPs). LNPs consist of mRNA encapsulated in ionizable lipids, phospholipids, cholesterol, and PEG-lipids. After cellular uptake, LNPs exit endosomes, release mRNA into the cytoplasm, enable translation, and induce CAR expression on the T cell surface.

Lipid nanoparticles (LNPs) are currently the most effective modality for systemic delivery of mRNA, and a standard LNP formulation comprises four lipid components, each serving a specific biophysical function [31]. The ionizable lipid, which is the most critical design element, contains a titratable amine group with an apparent pKa typically between 6.0 and 6.5. In this range, ionizable lipids remain predominantly neutral at physiological pH (7.4), thereby reducing non-specific electrostatic interactions and systemic toxicity. As the pH within the endosomal compartment drops to approximately 5.0–5.5, these amines become protonated, conferring a positive charge and promoting a “cone-shaped” lipid geometry that destabilizes the endosomal membrane and facilitates release of the mRNA cargo into the cytoplasm [32]. Helper phospholipids such as DOPE or DSPC provide the structural scaffold of the bilayer and modulate membrane fluidity. Cholesterol intercalates between phospholipids, fills packing defects, enhances particle stability, and has also been shown to support endosomal escape [33]. Poly (ethylene glycol) (PEG)-lipid conjugates coat the outer surface of LNPs, preventing particle aggregation, reducing opsonization, and extending circulation time by creating a hydrophilic steric barrier [34]. However, the PEG layer must be carefully calibrated, as excessive PEGylation can impair cellular uptake and endosomal escape, whereas insufficient PEG coverage can compromise colloidal stability and accelerate clearance [35]. The key components of the mRNA-LNPs for in vivo CAR-T generation are shown in Table 1. The structural organization and functional roles of each lipid component in mRNA-loaded lipid nanoparticles are summarized in Figure 3. 

### 2.3. Ionizable Lipid Evolution and Structure Activity Relationships

Ionizable lipid design has evolved rapidly since DLin-MC3-DMA (MC3) was clinically validated in Onpattro (patisiran), the first FDA-approved LNP-based nucleic acid therapeutic [36]. MC3 and its successors, including Moderna’s SM-102 (used in mRNA-1273) and Acuitas ALC-0315 (used in BNT162b2), have defined key structure–activity relationships for ionizable lipids in LNP formulations [37]. In general, branched lipid tails with cis-double bonds increase membrane fusogenicity, ester linkages promote biodegradability and reduce hepatotoxicity, and tail lengths of approximately 9–14 carbons help balance efficient cargo encapsulation with productive endosomal escape [38]. For T cell-targeted applications, the intrinsic hepatic tropism of conventional LNPs, driven in part by apolipoprotein E (ApoE) binding to low-density lipoprotein receptors, represents a major limitation that must be overcome [39]. Groundbreaking studies from the Weissman and Mitchell laboratories have shown that systematic modification of ionizable lipid chemistry, adjustment of lipid component ratios, and incorporation of so-called Selective ORgan-Targeting (SORT) lipids can shift LNP biodistribution away from the liver and towards lymphoid organs and circulating immune cells [40,41]. More recently, high-throughput barcoded LNP screening platforms (for example, fast identification of nanoparticle delivery, FIND) have accelerated the discovery of ionizable lipid architectures that preferentially transfect T lymphocytes in vivo, providing a rational framework for designing LNPs suited to in vivo CAR-T applications [42].

## 3. Engineering T Cell-Targeted LNPs

### 3.1. Challenge of T Cell Transfection

T lymphocytes pose substantial challenges for non-viral gene delivery. Resting T cells are small, metabolically quiescent, non-phagocytic cells with low levels of endocytic receptors, in marked contrast to rapidly dividing hepatocytes or tumor cells that are more permissive for uptake [43]. Their efficient DNA repair machinery and stringent control of nuclear import further impede plasmid DNA delivery, making stable nuclear transfection particularly inefficient in this lineage. By bypassing the requirement for nuclear entry, mRNA represents a more suitable cargo for T cell reprogramming [44]; nevertheless, therapeutic mRNA must still overcome the plasma membrane barrier and endosomal sequestration to reach the cytoplasm in a timely manner. Historically, electroporation has been used to transfect T cells in a virus-free manner and can achieve high transfection efficiencies, but it is associated with substantial cell stress and viability loss and inherently requires ex vivo manipulation [45]. The in vivo delivery problem is therefore more complex and demands formulation and targeting strategies that are tailored to T cell biology. For LNP-based approaches, effective T cell programming requires particles that (1) persist in the circulation long enough to encounter relevant T cell populations, (2) selectively recognize T lymphocytes through high-affinity interactions with surface markers such as CD3, CD4, CD5 or CD8, (3) undergo efficient receptor-mediated endocytosis into these cells and (4) achieve robust endosomal escape to deliver functional mRNA to the cytosol [45]. Early work with antibody-decorated LNPs and immunotropic lipids has shown that it is possible to bias biodistribution towards lymphoid tissues and T cell subsets in vivo, and several platforms now in preclinical and clinical development exploit such ligand-directed or tropism-based strategies to engineer T cells or T cell subsets directly in situ [46]. T lymphocytes pose significant obstacles to the delivery of non-viral nucleic acids. Resting T cells are small, inactive, non-phagocytic cells that have low levels of endocytic receptors. This is different from hepatocytes or tumor cells that divide quickly. Their effective DNA repair systems and limits on nuclear imports make it even harder to deliver plasmid DNA. mRNA does not have to go through the nuclear entry requirements, which makes it the best cargo for T cell reprogramming. However, to get it into the cytoplasm quickly, it still must get past the membrane’s impermeability and the endosomal sequestration. Electroporation was used in the past to transfect T cells without using viruses. It works well, but it is harmful to cells and requires manipulating cells outside of the body. The in vivo delivery problem requires LNPs that can (1) stay in the bloodstream long enough to meet T cells, (2) selectively bind to T lymphocyte surface markers, (3) be taken up by receptor-mediated endocytosis, and (4) get out of the endosome well enough to deliver functional mRNA to the cytoplasm [47].

### 3.2. Active Targeting Strategies

Anti-CD3 and anti-CD8 antibody conjugation: CD3 is the invariant signaling complex of the T cell receptor and is expressed exclusively on T lymphocytes, making it an attractive target antigen for active delivery. Groundbreaking work from the Bhatt and Alabi laboratories showed that LNPs functionalized with anti-CD3 single-chain variable fragments (scFvs) or Fab fragments are markedly more efficient at transfecting T cells, both in vitro and in vivo [48]. In humanized mouse models, CD3-targeted LNPs delivered mRNA to T cells up to ~30-fold more effectively than non-targeted particles [49]. Targeting CD8 similarly enables selective delivery to cytotoxic T lymphocytes, the principal effector cells in antitumor immunity. Because CD4^+^ helper T cells also contribute to tumor control and sustain CAR-T persistence, pan-T strategies that use binders against CD3 or CD2 are often preferred when broad T cell reprogramming is desired [50]. CD5 has also been explored as a target antigen; CD5-targeted LNPs efficiently transfect T cells in vivo and drive CAR expression at levels sufficient to induce antitumor activity in lymphoma models [20].

Nanobody and single-domain antibody approaches: Camelid single-domain antibodies (nanobodies and VHHs) are well suited for LNP surface functionalization owing to their small size (~15 kDa), high stability, and ease of genetic and chemical engineering [51]. Sortase-mediated ligation and maleimide–thiol coupling have been used to conjugate nanobodies specific for CD7, a pan-T cell marker that is highly expressed on both T cells and natural killer cells, to ionizable LNPs [22]. Following intravenous administration in mouse models, CD7-targeted mRNA–LNPs accumulated predominantly in T cells, and in xenograft settings, CAR expression from these particles was sufficient to drive tumor regression [21].

Receptor-specific ligands and LFA-1 targeting: Peptides and small molecules that bind T cell-restricted surface receptors provide an attractive alternative to antibody-based approaches, particularly from a manufacturing standpoint [52]. Lymphocyte function-associated antigen 1 (LFA-1), the integrin αL/β2 heterodimer abundantly expressed on T cells, has been exploited for selective LNP targeting using LABL, a peptide derived from ICAM-1 [53,54]. LABL-functionalized LNPs show preferential binding to and internalization by T cells, demonstrating that short peptide ligands can effectively tune LNP tropism without the need for full antibody conjugation [55].

### 3.3. Passive Targeting and SORT Technology

An additional, complementary strategy leverages the fact that LNP biodistribution can be modulated purely by adjusting lipid composition [56]. The Siegwart laboratory’s Selective ORgan Targeting (SORT) technology demonstrated that introducing a fifth lipid component, typically a permanently charged cationic or anionic lipid, into otherwise conventional four-component LNPs can systematically retune organ tropism, redirecting delivery to the lung, spleen, or liver depending on the identity and proportion of the SORT lipid. In this framework, spleen-directed SORT LNPs preferentially transfect splenic T lymphocytes, providing a relatively simple route to immune cell targeting that obviates the need for surface conjugation of antibodies or other exogenous ligands [41]. Another strategy exploits the possibility to modulate LNP biodistribution just by changing the lipid composition, without the need for exogenous targeting ligands. The SORT platform of the Siegwart laboratory demonstrated that the addition of a fifth lipid component to otherwise conventional four-component LNPs systematically retunes organ tropism in vivo. Permanently cationic SORT lipids, such as DOTAP, redirected delivery from the liver to the lung, permanently anionic lipids, such as 18PA, redirected tropism to the spleen, and additional ionizable amino lipids increased hepatic delivery. The effect was found to generalize across multiple ionizable lipid backbones and nucleic acid cargoes, including mRNA and CRISPR–Cas9 components. Additional mechanistic studies revealed that SORT lipids operate less by altering the overall surface charge and more by programming the in situ protein corona: anionic formulations selectively adsorb β2-glycoprotein I to recruit splenic phagocytes, cationic formulations adsorb vitronectin to target pulmonary endothelium, and hepatic variants maintain canonical ApoE-mediated uptake, reframing SORT as a type of proactive endogenous ligand targeting rather than simply passive distribution. For immunological applications, spleen-targeted anionic SORT LNPs preferentially transfect splenic B cells, T cells, and dendritic cells. Other research showed robust mRNA expression in splenic T lymphocytes after optimization of anionic SORT composition. This provides a simple route to lymphoid targeting that obviates antibody conjugation and has since enabled in situ engineering of regulatory T cells, mRNA vaccination, and CRISPR editing of hematopoietic compartments. SORT is particularly attractive for translation as it maintains the size range and manufacturing pipeline of conventional LNPs and avoids ligand-related immunogenicity. Although cell type selectivity within the targeted organ is not complete, rodent-derived biodistribution patterns may not translate directly to humans, given interspecies differences in plasma proteins.

## 4. CAR Construct Design for mRNA Delivery

### 4.1. Architecture of CAR Molecules

**Figure 4:**
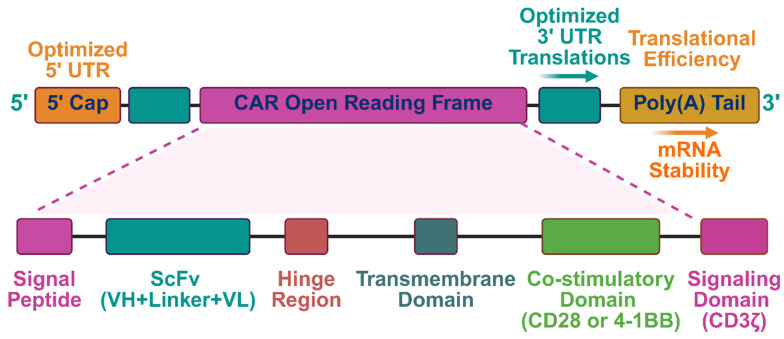
CAR mRNA construct and protein structure schematic. The mRNA contains an optimized 5′ UTR with 5′ cap, a CAR open reading frame, and an optimized 3′ UTR with a poly(A) tail for improved translational efficiency and stability. The CAR protein is encoded with a signal peptide, single-chain variable fragment (scFv; VH–linker–VL), hinge region, transmembrane domain, co-stimulatory domain (e.g., CD28 or 4-1BB), and CD3ζ signaling domain.

The canonical chimeric antigen receptor (CAR) is a synthetic type I transmembrane protein composed of four functional modules. The extracellular antigen-binding domain is usually derived from the variable-heavy (VH) and variable-light (VL) chains of a tumor-antigen-specific antibody configured as a single-chain variable fragment (scFv) [57]. A hinge or spacer region connects this binding domain to the membrane and provides flexibility, thereby influencing epitope reach and the optimal intercellular distance for target engagement [58]. The transmembrane domain anchors the receptor in the T cell membrane and can modulate receptor stability and signaling. Finally, one or more intracellular signaling domains transmit activation signals following antigen binding. The current clinical standard consists of second-generation CARs, which combine the CD3ζ primary signaling domain with a single co-stimulatory module, most commonly CD28 or 4-1BB (CD137) [59]. CD28-containing CARs drive rapid and robust effector function, whereas 4-1BB-containing CARs favor enhanced persistence and T cell fitness, in part by supporting mitochondrial biogenesis [60]. Third-generation CARs incorporate both co-stimulatory domains in tandem; however, this added complexity has not consistently translated into superior clinical outcomes. The structural organization of CAR-T is shown in Figure 4. For mRNA delivery, the intrinsically transient nature of CAR expression introduces design considerations that do not apply to constructs delivered via stable genomic integration [61].

### 4.2. mRNA-Specific CAR Design Considerations

Because mRNA is naturally short-lived, with effective half-lives on the order of 12–72 h even with nucleoside modification and 3′ UTR optimization, mRNA-encoded CARs provide a defined window of effector activity followed by spontaneous loss of expression [62]. This feature offers a clear safety advantage by eliminating the risk of insertional mutagenesis and long-term on-target off-tumor toxicity. At the same time, it creates a pharmacological challenge: sufficient CAR surface density must be achieved on T cells within the therapeutic window, and redosing strategies must be devised for tumors that require prolonged antigenic pressure [63]. Multiple approaches are being explored to extend both the duration and magnitude of mRNA-CAR expression. Circular RNA (circRNA) is intrinsically more stable than linear RNA because it lacks the 5′ cap and poly(A) tail that exonucleases typically recognize and can therefore support continuous protein production from a single delivery event [64,65,66]. Self-amplifying RNA (saRNA) encodes both the alphavirus replication machinery and the CAR transgene, enabling intracellular amplification of the RNA template and sustained protein expression from very small input doses, often over weeks [67,68,69]. In vivo CAR-T applications can, in principle, use both circRNA and saRNA as next-generation alternatives to conventional linear mRNA.

### 4.3. Safety Switches and Armored CARs

The transient expression profile of mRNA provides an inherent safety mechanism. Toxicities such as cytokine release syndrome (CRS) or on-target off-tumor effects are self-limiting as the mRNA degrades and CAR expression declines [70]. Nevertheless, additional safety switches have been incorporated into mRNA-CAR designs to allow active termination of CAR-T activity if needed. One strategy relies on the small molecule rimiducid (AP1903), which activates the inducible caspase 9 (iCasp9) suicide gene. Co-expression of iCasp9 with mRNA-encoded CARs enables selective elimination of CAR-T cells on demand [71]. The mRNA co-delivery strategies also facilitate “armored” CAR constructs in which immunomodulatory payloads are expressed alongside the CAR [72]. CAR designs have been modified to co-express cytokines such as IL-15 or IL-21, or dominant-negative TGF-β receptors, to enhance T cell proliferation, persistence, and resistance to immunosuppressive signals within the tumor microenvironment [73]. In the context of LNP delivery, simultaneous administration of CAR mRNA and mRNA encoding cytokines or checkpoint modulators (for example, PD-1 decoy receptors) can, in principle, remodel the local tumor microenvironment without inducing systemic toxicity, as these payloads remain confined to the transfected T cells [29].

## 5. Preclinical Evidence

### 5.1. Hematologic Malignancy Models

The concept of in vivo mRNA-CAR-T cell generation was first rigorously demonstrated in murine models of B cell malignancies [74]. In 2020, Parayath and colleagues showed that anti-CD3-targeted LNPs encapsulating CD19-CAR mRNA could program functional CAR-T cells in vivo in immunocompetent mice bearing A20 B cell lymphoma [48,75]. In that study, about 2–7% of circulating CD3+ T cells expressed detectable CAR protein 24 h after a single intravenous dose, while the bulk of the injected LNP dose accumulated in the liver and splenic phagocytes, a biodistribution pattern similar to that of intravenously administered LNPs regardless of surface ligand. Despite the modest T cell transfection fraction, treated animals showed significantly reduced tumor growth and prolonged survival [48]. CAR expression on circulating T cells was detectable within 24 h of a single intravenous dose, with expression declining over 5–7 days, consistent with the expected pharmacokinetics of an mRNA payload and without evidence of systemic toxicity [75]. Subsequent work extended these observations to human T cells in xenograft settings. In humanized NSG mice reconstituted with human peripheral blood mononuclear cells, anti-CD3-targeted LNPs loaded with CD19-CAR mRNA transfected roughly 15–17% of human CD3^+^ T cells in spleen and peripheral blood, again against a background of substantial hepatic and splenic myeloid uptake, and the resulting mRNA-engineered human CAR-T cells mediated antigen-specific cytotoxicity against CD19^+^ tumor targets [76]. Nonetheless, these reports should be read with quantitative caution. Across published in vivo CAR-T studies, a small minority of the total dose, on the order of single-digit to low double-digit percentages, even with optimized targeting ligands, remains internalized by T lymphocytes, with the bulk of particles cleared by hepatocytes and reticuloendothelial phagocytes. T cell transfection efficiencies have also been reported to range widely from <5% in earlier anti-CD3 systems to ~17–22% with CD5-targeted formulations and higher numbers claimed in some CD8-targeted or SORT-based platforms, but these numbers are not directly comparable across studies because they represent different denominators (CAR+/total T cells, CAR+/transfected cells or biodistribution based dose fraction), different time points and different organs sampled. Importantly, the overwhelming uptake of CAR mRNA by phagocytes and hepatocytes suggests that a large fraction of CAR mRNA is translated in non-T cell compartments. The functional consequences of this, such as off-target CAR signaling in macrophages, hepatic CAR expression, and potential contribution to cytokine release, have not been systematically characterized in most reports. Standardized reporting of (i) absolute and relative T cell transfection percentages, (ii) the fraction of total injected dose recovered in T cells versus other lineages, and (iii) CAR expression in off-target cells would go a long way to improving the interpretability and comparability of preclinical in vivo CAR-T studies going forward. In comparison, anti-CD3 targeted platforms and anti-CD5 targeted platforms show broadly similar CAR expression kinetics (~5–7 days) but differ in T cell subset bias, with CD5 targeted LNPs preferentially engaging naive and memory pools and CD3 targeted LNPs producing more uniform but transient activation. Reported transfection efficiencies in humanized NSG models also remain behind fully murine systems, and persistence data are inconsistent across studies, likely reflecting differences in the CAR construct, ionizable lipid chemistry, and dosing schedule, rather than a true biological discrepancy. Representative preclinical studies demonstrating in vivo mRNA-LNP CAR-T generation are shown in Table 2, while clinical LNP platforms are shown in Table 3.

### 5.2. Solid Tumor Applications

Hematologic malignancies have provided the earliest validation of in vivo mRNA–CAR-T approaches, but the most stringent test of this modality is in solid tumors, where conventional ex vivo CAR-T therapies have historically shown limited efficacy [81]. In this setting, CAR-T cells must contend with a profoundly immunosuppressive tumor microenvironment characterized by elevated TGF-β, IL-10 and PD-L1 expression, as well as abundant regulatory T cells and myeloid-derived suppressor cells, all of which impede T cell infiltration, persistence and effector function [82]. Preliminary data nevertheless indicate that in vivo-generated CAR-T cells can access solid tumor sites when delivered intratumorally or via appropriately engineered systemic formulations [83]. A notable example is the work by Billingsley and colleagues, who used LFA-1-targeted mRNA-LNPs to deliver GD2-CAR mRNA in a syngeneic neuroblastoma model [21,84]. Systemic (intravenous) administration of these particles generated CAR-T cells in vivo that could traffic to the tumor bed and exert antitumor activity, resulting in meaningful tumor control compared with non-targeted or control formulations [83]. Despite this activity, challenges with deep tumor infiltration and durable CAR-T persistence remained, underscoring the need for further optimization of both CAR design and delivery parameters in solid tumor indications [85]. A seminal 2022 study by Rurik and colleagues, although not conducted in an oncologic context, provided compelling proof-of-concept that in vivo mRNA-CAR-T cells can be directed against non-lymphoid targets [20]. In a murine model of heart failure, CD5-targeted LNPs were used to deliver FAP-CAR mRNA, leading to transient in vivo generation of CAR-T cells that selectively recognized and depleted cardiac fibroblasts [86]. This intervention reduced cardiac fibrosis and improved cardiac function, demonstrating that in vivo-programmed CAR-T cells can remodel pathogenic stromal compartments outside of the hematologic system. Beyond broadening the prospective therapeutic scope of in vivo CAR-T technology, this study highlighted the platform’s adaptability and translational potential in a physiologically rigorous model, helping to catalyze interest in applying in vivo mRNA-CAR-T strategies across a wider spectrum of solid and fibrotic diseases [87]. The results are much more heterogeneous across solid tumor studies than in hematologic models. Intratumoral delivery has consistently outperformed systemic administration in tumor cell killing, but with limited utility for disseminated disease; and systemically delivered LFA-1- or CD5 targeted LNPs have achieved tumor trafficking, but with variable and often modest CAR-T persistence. The fibrosis directed efficacy should also be interpreted with caution in extrapolation to oncologic settings, as cardiac fibroblast clearance and tumor stromal remodeling are subject to distinct microenvironmental barriers. Clinical rationale for selecting platforms across different disease contexts were summarized in Table 4, while Ongoing and emerging clinical studies evaluating in vivo mRNA–LNP CAR-T approaches are presented in Table 5.

### 5.3. Non-Human Primate Studies

Non-human primate (NHP) models represent a critical bridge between murine studies and human translation for in vivo mRNA-CAR-T therapies, because differences in LNP biodistribution, innate immune sensing, and T cell biology between rodents and primates can substantially influence both efficacy and safety [96]. Early NHP investigations with T cell-targeted mRNA-LNPs have shown that intravenously administered, ligand-decorated LNPs can achieve productive T cell transfection in macaques and cynomolgus monkeys, with acceptable tolerability and only transient cytokine elevations consistent with on-target pharmacodynamic activity [97]. Although much of this work remains incompletely described in peer-reviewed publications, the available data have been sufficient to support IND-enabling packages for several in vivo CAR-T programs and to justify progression toward first-in-human studies [98]. Successful translation of dosing regimens from mice to primates requires more than simple body-weight normalization [99]. Allometric scaling must be integrated with a detailed understanding of species-specific differences in T cell frequency and distribution, activation thresholds, and the molecular determinants of LNP recognition, including apolipoprotein interactions and complement pathways [100]. Current academic and industrial efforts, therefore, place strong emphasis on comparative biodistribution and pharmacology studies in NHPs, iterative optimization of ionizable lipid chemistry and PEG-lipid content, and high-throughput screening of targeted LNP libraries directly in primates [101]. These endeavors aim to establish dosing strategies and safety margins that more faithfully anticipate human responses, thereby de-risking clinical translation of in vivo mRNA–CAR-T platforms. One major caveat is that much of the evidence in NHP to date is in the form of conference abstracts or disclosures by sponsors rather than peer-reviewed reports. The publicly available datasets show appreciable variability in T cell transfection efficiency, magnitude of cytokine release, and complement activation between studies, even when nominally similar platforms are compared. These inconsistencies, in conjunction with reported species differences in apolipoprotein binding and TLR repertoires, argue against direct extrapolation of doses from rodents to primates and highlight the need for harmonized pharmacology endpoints prior to clinical translation.

## 6. Clinical Translation and Regulatory Landscape

The preclinical success of in vivo mRNA-CAR-T technologies has catalyzed substantial investment and early-stage clinical activity, particularly among biotechnology companies focused on targeted LNP platforms. Capstan Therapeutics, Sana Biotechnology (with its in vivo fusosome approach), and several academic spin-outs have disclosed IND-enabling programs and initiated first-in-human trials to test in vivo T cell reprogramming strategies [102,103,104]. Most of these programs are currently in the IND-enabling or phase I dose-escalation stage, but they collectively mark a shift from conceptual feasibility towards systematic clinical evaluation. Capstan Therapeutics, in particular, has highlighted the development of targeted mRNA-LNPs designed to generate CD19-directed CAR-T cells in vivo; preclinical data in humanized models demonstrate selective T cell transfection and robust antitumor activity, positioning these programs among the closest to a fully clinically translated in vivo mRNA-CAR-T product and illustrating the likely trajectory of the field [105]. The regulatory pathway for in vivo mRNA-CAR-T therapies draws on, but does not fully overlap with, existing frameworks for gene therapy products and ex vivo CAR-T cells. Under current U.S. and EU paradigms, LNP-mRNA formulations are regulated as biological products (for example, under 21 CFR biologics provisions), whereas the CAR-T cells that arise in vivo constitute a gene-modified somatic cell therapy [83]. This dual nature raises novel questions about how the “product” should be defined (vector only versus vector plus in vivo generated cells), what constitutes appropriate release criteria, and how potency should be assessed when the active cellular drug is created inside the patient. Regulatory agencies such as the FDA’s Center for Biologics Evaluation and Research (CBER) and the European Medicines Agency (EMA) are beginning to address these issues through guidance updates and regulatory science initiatives, often by analogy to in vivo gene therapy and traditional CAR-T frameworks. Core expectations include: demonstration of consistent LNP manufacturing (particle size distribution, encapsulation efficiency, mRNA integrity, and targeting-ligand conjugation efficiency); detailed characterization of the phenotype and function of in vivo-generated CAR-T cells in patient samples; quantitative modeling of the pharmacokinetics and pharmacodynamics of CAR expression and expansion; and comprehensive safety assessment, including genotoxicity and insertional risk even for non-integrating mRNA platforms, to robustly exclude unintended nuclear delivery or off-target genome modification [106].

### Biomarkers and Clinical Monitoring

Because in vivo mRNA-CAR-T products cannot be functionally tested before infusion in the same way as ex vivo manufactured cell products, real-time pharmacodynamic monitoring becomes central to risk management and dose optimization [107]. For clinical development, there is a growing emphasis on developing and standardizing biomarkers for in vivo CAR-T monitoring, drawing on experience from ex vivo CAR-T trials [108]. Flow cytometry and quantitative or digital PCR remain core tools for detecting CAR-positive T cells and quantifying their expansion and persistence in peripheral blood [109]. In the in vivo context, additional candidate biomarkers include soluble CAR fragments, CAR-associated cytokine signatures, and imaging-based methods capable of tracking CAR-T biodistribution and activity in tissues, which together may provide a more complete picture of on-target engagement and emerging toxicities [110]. Regulators and developers are also converging on the need for long-term follow-up, like ex vivo CAR-T and gene therapy products, to capture delayed adverse events, secondary malignancies and durability of response [106,111]. As clinical data accumulate, these insights are expected to inform refined classification schemes, potency assays and adaptive trial designs specific to in vivo mRNA-CAR-T therapies, ultimately enabling regulatory pathways that balance accelerated access with rigorous safety evaluation.

## 7. Challenges and Limitations

### 7.1. Specificity and Off-Target Transfection

Achieving sufficient cell specificity is arguably the central challenge for in vivo mRNA-CAR-T therapy [63]. Current T cell-targeted LNPs show clear preferential uptake by T lymphocytes, yet they consistently transfect non-target cells to varying degrees [22]. Hepatocytes, professional antigen-presenting cells, natural killer cells, and other innate immune cells can internalize targeted LNPs via Fc receptor engagement, non-specific endocytosis, or bystander uptake [112]. Expression of CARs on non-T cells raises the possibility of aberrant cytotoxicity or inflammatory activation; however, the transient nature of mRNA expression limits the persistence of such off-target effects compared with integrating vectors [70]. An additional concern is inadvertent CAR expression in regulatory T cells, which could dampen anti-tumor immunity rather than augment it. To address these issues, multiple strategies are being explored to bias in vivo transfection towards cytotoxic CD8^+^ T cells and away from suppressive T cell subsets [113]. These include CD8-selective targeting, dual-marker strategies that favor effector populations, and formulation elements designed to reduce Treg uptake [114,115,116]. More broadly, further refinement of targeting ligands, linker chemistries, and LNP composition, as well as high-throughput screening in human primary cells and in vivo models, will be required to narrow the functional transfection window to the desired T cell pool while minimizing clinically relevant off-target expression [117].

### 7.2. Immunogenicity of LNPs and mRNA

Despite extensive nucleoside modification, mRNA-LNP formulations continue to engage innate immune sensors, including TLR-independent cytosolic RNA receptors such as RIG-I and MDA5, as well as the NLRP3 inflammasome [118]. Because CAR expression from mRNA is transient, most in vivo platforms will require repeated dosing, which raises concerns about cumulative inflammatory toxicity and the development of anti-LNP immunity over time [119]. Repeated administration of PEGylated nanoparticles can trigger the “accelerated blood clearance” (ABC) phenomenon, in which anti-PEG antibodies drive faster LNP clearance and enhanced complement-mediated uptake on subsequent injections [120]. Anti-PEG antibodies are detectable in approximately 40–72% of the general population, largely due to prior exposure to PEG-containing products, and can substantially shorten LNP circulation time and alter biodistribution in pre-sensitized individuals [111,121]. These observations have stimulated efforts to develop alternative “stealth” strategies that circumvent pre-existing anti-PEG immunity [122]. Approaches under investigation include PEG-free coatings based on zwitterionic lipids, polysarcosine or related hydrophilic polymers, as well as incorporation of “don’t-eat-me” signals such as CD47 to attenuate phagocytic clearance [123,124,125,126,127]. In parallel, rational tuning of ionizable lipid chemistry and LNP composition aims to reduce innate reactogenicity while preserving or even enhancing delivery to target T cells [128]. For in vivo mRNA-CAR-T applications that may require cyclical dosing, careful characterization of anti-lipid and anti-PEG responses, complement activation, and cytokine profiles will be essential components of clinical development and risk management [21,129].

### 7.3. Manufacturing and Scalability

**Figure 5:**
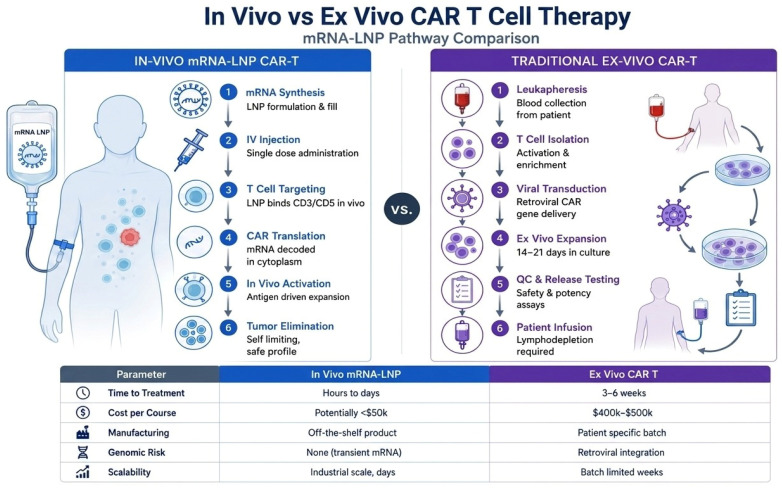
In vivo mRNA-LNP versus ex vivo CAR-T cell therapy. Schematic comparison of workflows and key attributes. (**Left**) In vivo CAR-T is generated by intravenous delivery of mRNA-LNPs that transiently express CARs in T cells, enabling antigen-driven activation and tumor clearance without genomic integration. (**Right**) Ex vivo CAR-T involves leukapheresis, viral gene transfer, expansion, and reinfusion following lymphodepletion. (**Bottom**) Major differences in time to treatment, cost, manufacturing (off-the-shelf vs patient-specific), genomic risk, and scalability are summarized.

Although in vivo mRNA-CAR-T therapy obviates patient-specific cell manufacturing, it introduces a different layer of complexity at the level of RNA and nanoparticle production. Generation of high-quality, GMP-grade mRNA typically involves in vitro transcription from linearized plasmid DNA templates, followed by purification (often using HPLC) to remove double-stranded RNA contaminants, incorporation of modified nucleosides, and rigorous quality control to confirm sequence identity, cap integrity, poly(A) tail length, and the absence of residual DNA. Clinical-scale LNP manufacturing requires precise, scalable mixing technologies such as microfluidic systems, tight control of particle size and polydispersity, optimization of encapsulation efficiency, and comprehensive characterization of stability and potency over the product life cycle [111]. Targeting-ligand conjugation, particularly for antibody-functionalized LNPs, adds further complexity. Conjugation efficiency must be consistent across batches, unconjugated antibody must be removed, and the conjugation process must preserve both antibody binding affinity and LNP structural integrity [130]. Established bioconjugation platforms can help standardize these steps, but they increase manufacturing cost and process sophistication relative to untargeted LNPs, and they introduce additional critical quality attributes that must be monitored and controlled under GMP. As in vivo CAR-T products advance, early integration of formulation and process development, and close alignment between chemistry manufacturing controls (CMC) strategy and regulatory expectations, will be pivotal for scalable, reproducible production [48]. Importantly, a third configuration, ex vivo electroporation or LNP-mediated mRNA transfection of patient T cells, is somewhat intermediate between permanent DNA-modified CAR-T (retroviral, lentiviral, or in vivo lentiviral LNP delivered) and fully in vivo mRNA-LNP CAR-T. Like the in vivo mRNA approach, ex vivo mRNA modification results in transient, self-limiting CAR expression and thereby lowers the risks of insertional mutagenesis and long-term off-tumor toxicity; however, it still has the expense, time, and logistical burden of autologous cell manufacturing and lymphodepletion, and therefore addresses the safety/permanence issue but does not solve the accessibility limitations that drive in vivo platforms. A comparison between ex vivo and in vivo CAR-T cell engineering approaches is shown in Figure 5 and Table 6.

### 7.4. Tumor Microenvironment Barriers

The immunosuppressive tumor microenvironment (TME) remains a major obstacle for in vivo–generated CAR-T cells, mirroring the challenges seen with ex vivo products [131]. CAR-T cells arising from systemic LNP delivery must function within a milieu characterized by PD-1/PD-L1 checkpoint engagement, TGF-β–driven exhaustion, adenosine-mediated anergy and intense metabolic competition with tumor and stromal cells [81]. These factors collectively limit CAR-T cell infiltration, survival and cytotoxicity in solid tumors. Combination strategies are therefore emerging as a key focus for in vivo mRNA–CAR-T platforms [132]. Co-administration of CAR mRNA with mRNA-LNPs encoding checkpoint inhibitors, metabolic modulators or cytokines (for example, IL-12, IL-15 or IL-21) offers a means to locally reshape the TME and enhance CAR-T function while minimizing systemic exposure to potent immunostimulatory agents [133]. Similarly, “armored” CAR designs that co-express dominant-negative receptors (such as TGF-β receptor variants) or PD-1 decoy receptors via mRNA co-delivery could confer resistance to key suppressive pathways in situ [85]. Integrating these approaches with rational antigen selection, trafficking cues and dosing strategies will be essential to fully unlock the potential of in vivo mRNA-CAR-T therapies in solid tumors [134].

## 8. Future Perspectives

### Multi-Antigen and Logic-Gated CARs

Next-generation CAR architectures for mRNA delivery are moving beyond single-antigen targeting. Dual-targeting CARs implemented either as tandem scFv constructs or as split “AND-gate” designs that require concurrent recognition of two tumor antigens can reduce the risk of antigen escape and improve tumor selectivity [111,135]. SynNotch systems, in which recognition of a priming antigen induces expression of a second receptor, can be encoded across two co-delivered mRNA species, enabling Boolean logic operations such as CAR-T cells that exert cytotoxicity only when both antigen A and antigen B are present [136]. The inherently short-lived nature of mRNA-encoded receptors is well suited to such logic-gated and safety-switch architectures, because any unintended activity is self-limiting as the transcripts decay [137,138].

CRISPR mRNA combinations: The simultaneous delivery of Cas9 mRNA and guide RNAs together with CAR mRNA offers the prospect of in vivo gene editing alongside CAR installation, without the need for viral vectors [139]. In principle, this approach could be used to disrupt endogenous T cell checkpoints (such as PD1, CTLA4, and LAG3) at the same time as introducing a CAR, thereby generating CAR-T cells with enhanced persistence and reduced exhaustion directly within the patient [140]. This would represent a convergence of non-viral gene delivery and precision immunoengineering in a single therapeutic platform. Initial in vitro studies have shown that concurrent CRISPR editing and CAR mRNA expression in T cells is feasible, and ongoing work is exploring whether analogous strategies can be safely and efficiently applied in vivo [141].

Universal and off-the-shelf considerations: One of the most transformative possibilities of in vivo mRNA-CAR-T therapy is the creation of truly off-the-shelf products: single LNP formulations manufactured at scale, stored as ready-to-use vials, and administered to any eligible patient without bespoke cell processing [142]. Realizing this vision will require demonstrating that CAR-T cells generated in vivo from a patient’s own T cells can achieve efficacy comparable to ex vivo-manufactured products across diverse clinical contexts, including advanced age, heavy prior treatment, and compromised immune competence, all factors that currently constrain the success of autologous manufacturing [17]. If these hurdles can be addressed, in vivo mRNA-CAR-T platforms could decouple access from specialized cell therapy infrastructure and substantially broaden the patient population that benefits from CAR-based treatments [143].

Beyond oncology: The cardiac fibrosis study by Rurik and colleagues illustrates that in vivo mRNA-CAR-T technology is not restricted to oncology. In that model, transient in vivo generation of FAP-directed CAR-T cells targeting cardiac fibroblasts reduced fibrosis and improved cardiac function, providing proof-of-concept for targeting pathogenic stromal cells in non-malignant disease [20]. More broadly, transient in vivo CAR-T cells directed against CD19 or BCMA could be used to deplete pathogenic B cells or plasma cells in autoimmune diseases such as systemic lupus erythematosus [20], myasthenia gravis [144], and pemphigus vulgaris [145], without the long-term engraftment concerns associated with permanently integrating CAR platforms [146]. Additional non-oncological applications under exploration include liver fibrosis (for example, FAP^+^ hepatic stellate cells), clearance of senescent cells, and control of chronic viral infections, highlighting the potential of in vivo mRNA-CAR-T approaches as a broadly applicable modality for immune-mediated tissue remodeling [147].

## 9. Conclusions

In vivo mRNA-CAR-T cell therapy sits at a genuine inflection point between scientific aspiration and clinical reality. In this emerging modality, advances in lipid nanoparticle (LNP) engineering, precision immune-cell targeting, synthetic biology, and mRNA therapeutics are converging to turn the patient’s own body into a CAR-T manufacturing bioreactor, eliminating the need for leukapheresis, ex vivo processing, and integrating viral vectors. Robust preclinical data across hematologic and solid tumor models, as well as non-oncology indications such as cardiac fibrosis, have established proof-of-concept for in vivo generation of functional CAR-engineered immune cells with meaningful antitumor or disease-modifying activity [17]. Early clinical programs using T cell-targeted and myeloid-tropic mRNA-LNP platforms now extend this paradigm into patients, providing the first evidence that in vivo mRNA-CAR technologies can achieve pharmacologically relevant CAR expression and target-cell depletion in humans [98]. Substantive obstacles remain in achieving high T cell specificity while minimizing off-target transfection, managing LNP and mRNA immunogenicity under repeat dosing, overcoming the immunosuppressive tumor microenvironment, and fitting this hybrid product (vector plus in vivo-generated cells) into existing regulatory frameworks are all non-trivial challenges [148]. Nonetheless, they are fundamentally engineering and translational science problems, for which conceptual and often experimental solutions already exist, refined targeting ligands and tropic lipids to sharpen cellular specificity, alternative “stealth” coatings and ionizable lipid chemistries to mitigate innate and anti-PEG responses, combinatorial or logic-gated payloads to counteract local immunosuppression, and adaptive regulatory paradigms that borrow from both gene therapy and cell therapy playbooks [149]. If these hurdles can be systematically addressed, the therapeutic prize is considerable as truly off-the-shelf, rapidly manufacturable and inherently self-limiting cell therapy, deliverable as a vial of RNA-LNP that can be administered to virtually any eligible patient, irrespective of age, prior treatment intensity, or logistical access to apheresis and GMP cell-processing infrastructure [150]. Such a shift would not only broaden the reach of CAR-based interventions across oncology, autoimmunity, and fibrotic or infectious diseases but also fundamentally change the economics and scalability of personalized immunotherapy. As the first wave of in vivo mRNA-CAR-T trials matures, the broader paradigm of in vivo synthetic immunology programming immune cells directly within the patient using non-viral nucleic acid technologies seems poised to become a defining force in the next decade of immuno-oncology and immune-modulating therapies.

## Figures and Tables

**Table 1:** Key components of mRNA-LNPs for in vivo CAR-T cell generation.

Component	Examples	Primary Function	Optimization Considerations
Ionizable Lipid	Dlin-MC3-DMA, SM-102, ALC-0315, Lipid5	mRNA encapsulation, endosomal escape	pKa 6.0–6.5, biodegradability, potency
Helper Phospholipid	DOPE, DSPC, DOPC	Bilayer structure, fluidity	Fusogenicity, immune activation
Cholesterol	Cholesterol, βsitosterol	Stability, endosomal escape	Percentage affects uptake efficiency
PEG-Lipid	PEG-DMG, PEG-DSPE	Steric stabilization, circulation time	Mol% and lipid anchor chain length
Targeting Ligand	Ani-CD3, anti-CD5, CD7 nanobody, LFA-1 ligand	T cell selective binding	Conjugation chemistry, receptor density

**Table 2:** Key preclinical studies of mRNA-LNP in vivo CAR-T cell generation.

LNP Targeting	CAR Target	Model	Key Finding	Limitation	References
Anti-CD3 scFv	CD19	Humanized NSG mice	Sustained B cell aplasia; tumor regression	Human-mouse LNP cross reactivity	[74,77]
Anti-CG3 Fab	CD19	Syngeneic A20 lymphoma	Complete remission in 7/9 mice	Transient CAR expression	[75]
CD5 targeted	CD19	NSG xenograft	T cell transfection; anti-tumor efficacy	Off-target NK cell transfection	[78]
CD5 targeted	FAP (fibroblasts)	Cardiac fibrosis model	First non-oncology in vivo CAT-T application	CAR-T used for fibrosis, not tumors	[20]
LFA-1 ligand	GD2	Syngeneic neuroblastoma	Selective CTL transfection; tumor control	Variable inter-animal efficacy	[79]
SORT technology	CD19	B-ALL mouse model	Spleen selective delivery; CAR expression	No NHP data presented	[80]

**Table 3:** LNP-based in vivo CAR platforms in clinical Studies.

Platform/Sponsor	LNP Type	Target Cell(s)	Payload Type	Lead CAR Targets	Indications (Stage)	Key Features/Comments
Capstan Therapeutics -CPTX2309	Antibody-decorated targeted LNPs with biodegradable, low-reactogenic ionizable lipids	CD8^+^ T cells (via anti-CD8 antibody)	Linear mRNA	CD19	B cell-mediated autoimmune diseases (phase I)	Two-dose intravenous regimen generates transient CD8^+^ CAR-T cells, deep B cell depletion, and immune reset in non-human primate and humanized models; designed for repeat dosing and minimized on-target toxicity.
Immorna-JCXH-213	Targeted lipid complex nanoparticles with mixed T/NK/myeloid tropism	T cells, NK cells, macrophages	Linear mRNA	CD19	CD19^+^ malignancies and immune diseases (phase I)	Seeks pharmacological synergy by co-reprogramming multiple effector compartments in vivo; first human dosing in 2025.
MagicRNA-HN2301	Encapsulating LNP platform with CD8-binding antibody fragments	CD8^+^ T cells	Linear mRNA	CD19	Relapsing/refractory systemic lupus erythematosus (phase I)	First disclosed human data for CD8-targeted LNP mRNA in vivo CAR-T in autoimmunity; demonstrates B cell depletion, cytokine signatures, and biomarker improvement without lymphodepletion.
Myeloid Therapeutics- MT-302/MT-303	Immunotropic LNPs favoring myeloid uptake	Monocytes/macrophages	Linear mRNA	TROP2, GPC3 (HER2, gp75 in preclinical work)	TROP2^+^ epithelial tumors and GPC3^+^ hepatocellular carcinoma (phase I)	Exploit natural LNP tropism for myeloid cells; generate CAR-macrophages that infiltrate tumors, phagocytose, and orchestrate broader immunity with manageable cytokine release syndrome.
Orna Therapeutics	LNPs selected for pan-T immunotropic lipids.	T cells/lymphocytes	Circular RNA	CD19	B cell malignancies and autoimmunity (preclinical)	Circular RNA extends CAR expression and activity relative to linear mRNA; LNPs tuned for repeat dosing and reduced innate reactogenicity.
Sanofi-LN15 and related LNPs	T cell–targeted LNPs with small binders (for example, anti-CD8 VHH)	T cells (for example, CD8^+^)	Linear mRNA	CD22, CD19	B cell malignancies and autoimmunity (preclinical to early clinical)	LN15 shows efficient T cell transfection, low liver uptake, and sustained expression over several days in preclinical models; now moving into clinical testing.
Academic CD5-LNP (Rurik and colleagues)	CD5-targeted LNPs	T cells (CD5^+^)	Linear mRNA	FAP	Cardiac fibrosis (preclinical)	Demonstrates that LNP-mediated in vivo CAR-T can remodel fibrotic tissue and restore organ function, providing a blueprint for non-oncology indications.
Academic LFA-1-LNP (Billingsley and colleagues)	LNPs functionalized with LABL peptide (LFA-1 ligand)	T cells with high LFA-1	Linear mRNA	GD2	Syngeneic neuroblastoma (preclinical)	Intravenous GD2-CAR LNPs generate tumor-homing CAR-T cells and control tumor growth, illustrating feasibility and residual tumor microenvironment barriers.
Peptide-LNP PSMA trial	Peptide-decorated LNPs targeting PSMA or T cell receptors	T cells	Linear mRNA	PSMA	Metastatic solid tumors (early phase I)	The first clinical example is that LNP-CAR mRNA can transiently reprogram circulating T cells in patients and produce measurable antitumor responses.

**Table 4:** Efficacy and disease scope of traditional ex vivo CAR-T versus in vivo mRNA-LNP CAR-T.

Disease Context	Traditional CAR-T	In Vivo CAR-T
Hematologic malignancies	Proven curative potential [88,89]	Emerging, unproven durability [75,90]
Solid tumors	Poor efficacy due to limited tumor infiltration, hostile microenvironment, and antigen heterogeneity [91,92]	Potential for enhanced tumor infiltration via in situ T cell engineering and repeated dosing [93]
Autoimmune disease	Risky due to permanent depletion [94,95]	Well-suited due to reversibility [20]
Acute indications	Often too slow to deploy [13]	Ideal for rapid intervention [20]

**Table 5:** RNA-based in vivo CAR platforms.

Company/ Group	RNA Format	Targeting Strategy	Engineered Cell Type(s)	Example CAR Targets	Indications (Stage)	Notes
Myeloid Therapeutics	Linear mRNA	Intrinsically myeloid-tropic LNPs (lipid tropism)	Monocytes/macrophages in blood and tumor microenvironment	TROP2, GPC3, HER2, gp75	MT-302 (TROP2^+^ epithelial tumors, phase I), MT-303 (GPC3^+^ hepatocellular carcinoma, phase I)	First clinical mRNA–LNP in vivo CAR programs; selective myeloid CAR expression, tumor infiltration, lesion reductions, acceptable safety, and manageable cytokine release syndrome.
Capstan Therapeutics (CellSeeker)	Linear mRNA	Antibody-decorated targeted LNPs (for example, anti-CD5, anti-CD8)	T cells (subset-directed, especially CD8^+^)	CD19 (lead), FAP (preclinical cardiac fibrosis)	CPTX2309 (anti-CD19, CD8^+^-targeted LNP, B cell–mediated autoimmunity, phase I)	Mouse and non-human primate data show rapid in vivo CD8^+^ CAR-T generation and deep but transient B cell depletion with compact two-dose regimens; transient mRNA expression leveraged for “immune reset” in autoimmunity.
Immorna Biotherapeutics	Linear mRNA	Targeted LNPs with tropism for T cells, NK cells and myeloid cells	Mixed immune cells (T, NK, myeloid)	CD19	JCXH-213 (CD19^+^ malignancies and immune diseases, phase I)	Co-reprograms multiple effector lineages in vivo; first patient dosed in 2025; positioned as first multi-immune-cell mRNA in vivo CAR therapy.
Shenzhen MagicRNA (Enc-LNP)	Linear mRNA	CD8-targeted LNPs with antibody fragments and proprietary ionizable lipids	CD8^+^ T cells	CD19 (HN2301)	Relapsing/refractory systemic lupus erythematosus, phase I	Early data show in vivo CD19 CAR-CD8^+^ T cell generation, B cell depletion and autoantibody decline without lymphodepletion, supporting an in vivo immune-reset paradigm.
Orna Therapeutics	Circular RNA (circRNA)	Immunotropic lipids (pan-T tropism)	T cells/lymphocytes	CD19	B cell malignancies and autoimmune diseases (preclinical; first-in-human planned)	Circular RNA prolongs CAR expression and activity versus linear mRNA and improves antitumor efficacy in preclinical models; positioned as next-generation mRNA format for in vivo CAR-T.
Sanofi (LN15 platform)	Linear mRNA	T cell–targeted LNPs (for example, anti-CD8 VHH)	T cells (for example, CD8^+^)	CD22, CD19	B cell malignancies and autoimmunity (preclinical to early clinical)	Preclinical mouse data show efficient T cell transfection, low liver uptake, and multi-day CAR expression; in vivo mRNA CAR-T program now entering clinical testing.
Academic (Rurik and colleagues)	Linear mRNA	CD5-targeted LNPs	T cells (CD5^+^ subsets)	FAP (cardiac fibroblasts)	Cardiac fibrosis in Murine heart failure models (preclinical)	Landmark proof of concept: transient in vivo CAR-T generation reduces fibrosis and restores cardiac function, expanding in vivo mRNA–CAR-T beyond oncology.
Academic (Billingsley and colleagues)	Linear mRNA	LFA-1-targeted LNPs (for example, LABL peptide)	T cells with high LFA-1	GD2	Syngeneic neuroblastoma model (preclinical)	Intravenous GD2-CAR mRNA LNPs generate CAR-T cells that traffic to solid tumors and control growth but highlight ongoing challenges in deep infiltration and persistence.
Other academic/early industry (PSMA, peptide-LNPs)	Linear mRNA	Peptide-functionalized LNPs (for example, PSMA- or T cell–targeting peptides)	T cells	PSMA and other tumor antigens	Metastatic solid tumors (early phase I PSMA CAR-mRNA LNP trial)	Show short-term but functionally effective CAR expression in circulating T cells and antitumor activity; represent early clinical translation of peptide-LNP in vivo CAR-T.

**Table 6:** Comparative overview of in vivo mRNA-LNP CAR-T and alternative engineered cell therapy modalities.

Modality	Engineering Mechanism	Key Advantages	Key Limitations
In vivo mRNA-LNP CAR-T	Transient mRNA expression of CAR in endogenous T cells via targeted LNPs	No ex vivo manufacturing or lymphodepletion; inherently self-limiting; repeat dosing feasible; rapid turnaround	Transient CAR expression may limit durable responses; cell type selectivity within target organ incomplete; long-term safety data lacking
Viral in vivo CAR delivery	Stable (lentivirus) or episomal (AAV) CAR transgene delivery directly to T cells in vivo	Durable CAR expression; potential single-dose therapy; demonstrated in NHPs	Insertional mutagenesis risk, pre-existing anti-vector immunity; complex and costly manufacturing; limited re-dosing
In vivo CRISPR engineering	LNP or AAV delivered Cas9/sgRNA for gene knockout or knock-in of CAR loci	Permanent genome modification; potential one-time treatment; validated for non-CAR indications	Off-target editing concerns; difficulty achieving site-specific CAR knock-in efficiency in vivo; immunogenicity of bacterial Cas proteins
Allogeneic/off-the-shelf CAR-T	Healthy donor T cell edited (TCR/HLA knockout) and expanded for universal use	Immediate availability; standardized manufacturing; lower cost per dose at scale	Host versus graft rejection limits persistence; intensive lymphodepletion required; mixed clinical efficacy vs autologous
CAR-NK cells	Allogeneic or iPSC-derived NK cells engineered with CAR	Low GVHD risk; innate cytotoxicity complements CAR signaling; off-the-shelf compatible	Shorter in vivo persistence than T cells; limited efficacy in heavily pretreated solid tumors; cryopreservation challenges
CAR-macrophages	Engineered macrophages with phagocytic CAR	Penetrate solid tumors; remodel tumor microenvironment; antigen spreading via cross presentation	Limited proliferation in vivo; manufacturing scalability constraints; early-stage efficacy data

## Data Availability

No data were used for the research described in the review.
